# Adaptation and Validation of a Treatment Expectations Scale for Hospitalized Patients-Spanish Patient Version

**DOI:** 10.3390/healthcare13162067

**Published:** 2025-08-21

**Authors:** Karol Gonzales-Valdivia, Katherine Ñaupa-Tito, Wilter C. Morales-García

**Affiliations:** 1 Escuela de Medicina Humana, Facultad de Ciencias de la Salud, Universidad Peruana Unión, Lima 15033, Peru; 2 Dirección General de Investigación, Universidad Peruana Unión, Lima 15033, Peru; 3 Escuela de Posgrado, Universidad Peruana Unión, Km 19, Carretera Central, Lima 15033, Peru

**Keywords:** hospitalization, expectations, patient, physician–patient relationships, quality of healthcare

## Abstract

*Background:* Hospitalized patients’ expectations about their treatment play a key role in therapeutic adherence, satisfaction with care, and clinical outcomes. However, there is a lack of brief, psychometrically validated instruments in Spanish-speaking contexts that adequately assess this construct. *Objective*: The objective of this study is to culturally adapt and validate the Hospitalized Patients’ Expectations for Treatment Scale-Patient Version (HOPE-P) in a Peruvian population. *Methods:* A methodological, cross-sectional study was conducted with 277 hospitalized patients aged 18 to 85 years (M = 45.87; SD = 17.09). The adaptation process included translation, back-translation, expert review, and pilot testing. Confirmatory factor analysis (CFA) was performed to assess the factor structure, and reliability and validity indices were calculated. *Results:* The bifactorial model showed good fit (CFI = 0.97, TLI = 0.94, RMSEA = 0.06). One item with a low factor loading was removed to improve the model. Convergent and discriminant validity were confirmed through acceptable values of Average Variance Extracted (0.60 and 0.55) and inter-factor correlation (φ^2^ = 0.23). Internal consistency was strong for both dimensions (α = 0.76–0.77; ω = 0.76–0.77). *Conclusions:* The Spanish version of the HOPE-P is a valid, reliable, and culturally appropriate instrument for evaluating treatment expectations in hospitalized Peruvian patients. Its implementation in clinical settings could enhance physician–patient communication, support shared decision-making, and contribute to better therapeutic outcomes, especially in high-demand healthcare environments.

## 1. Introduction

The expectations that hospitalized patients have regarding their treatment are a key factor in the clinical setting, influencing aspects related to healthcare economics, therapeutic outcomes, and patient safety. These expectations serve as important predictors in surgeries, experimental therapies, and pharmacological treatments, playing an essential role in patient satisfaction and treatment adherence [1,2,3,4]. For instance, preoperative expectations have been shown to significantly affect the length of hospital stay following procedures such as total hip arthroplasty. Patients with positive expectations tend to recover faster and experience fewer complications, highlighting the importance of setting realistic goals before surgery [2,5]. In psychiatry, initial expectations have also been found to influence the effectiveness of medications for major depression [6,7]. Similarly, in neurology, expectations are crucial to the outcomes of deep brain stimulation in Parkinson’s patients, improving both motor function and quality of life [8,9].

The placebo effect, closely linked to patient expectations, is a widely studied phenomenon across various medical fields. Positive initial expectations are associated with better psychotherapy outcomes, reinforcing the idea that patients’ beliefs about treatment can enhance its benefits [10,11]. This is also observed in dermatological conditions such as inflammatory dermatoses, where expectations influence treatment efficacy [12]. However, not all medical areas exhibit a uniform relationship between expectations and outcomes. For example, in edentulous older patients treated with dentures, initial expectations did not significantly impact their oral health-related quality of life [13,14]. Likewise, in conservatively treated radial fractures, expectations did not affect wrist function, underscoring the need to consider specific clinical characteristics when evaluating the role of expectations [15,16].

Expectations also directly affect patient safety and the perception of side effects. Low expectations can reduce treatment adherence, negatively impacting clinical outcomes [16,17]. Conversely, unrealistic expectations, such as the belief in an “immediate cure,” are associated with poorer disease control and lower treatment satisfaction [18]. In bariatric surgery, overestimating expected weight loss increases the risk of postoperative complications, emphasizing the importance of properly managing patient expectations [19]. Additionally, expectations play a role in the experience of side effects. For instance, anticipatory nausea before chemotherapy is a significant predictor of severe nausea afterward, highlighting the need to address expectations as part of clinical interventions [20,21]. Similarly, patients’ expectations toward their physicians are fundamental to the quality of care. Patient-centered care and shared decision-making have emerged as key approaches to improving physician–patient relationships and clinical outcomes [22].

Physicians’ beliefs and preferences significantly influence the therapeutic strategies they adopt, impacting not only perceptions of success but also patient satisfaction and adherence [23,24,25]. However, a communication gap between physicians and patients regarding expectations can lead to dissatisfaction and suboptimal outcomes [26,27,28]. This misalignment underscores the importance of strategies such as shared decision-making to align expectations and enhance mutual trust. Moreover, medical expectations can influence the patient’s physiological response. Studies have demonstrated that positive expectations from healthcare providers can modulate the release of neurochemicals such as endorphins, improving pain perception and enhancing healing responses [3,29]. This interaction highlights the need for a biopsychosocial approach to healthcare [30,31]. Finally, fostering realistic expectations through clear communication can significantly reduce patient stress and anxiety, improving their hospitalization experience and clinical outcomes [32,33].

Given the significance of patient expectations in clinical outcomes and safety, various instruments have been developed to comprehensively assess them. Existing scales are often designed for specific conditions, such as implantable cardioverter defibrillators [34], orthodontic treatments [35,36], or gynecological therapies [37]. While these tools are useful, they present limitations in high-demand clinical settings due to their length and lack of adaptability. For instance, general scales with up to 30 items may be impractical in hospital environments where time is limited [38]. In this context, the HOPE-P scale has emerged as a promising tool. Developed in China [39], it measures expectations related to physician–patient communication. It was applied to 210 hospitalized patients across departments such as gynecology, immunology, endocrinology, neurology, and cardiology. The critical ratio (CR) values for items 1–8 ranged from 6.036 to 8.354, showing significant differences between high- and low-scoring groups, while item 9 exhibited a non-significant negative correlation (−0.096). Exploratory factor analysis (EFA) validated a two-factor model—physician–patient communication and treatment outcome expectations—leading to the elimination of item 9 due to its negative factor loading and inadequate item–total correlation, which was further confirmed through confirmatory factor analysis (CFA). Ultimately, the instrument demonstrated high internal consistency and satisfactory test–retest reliability, resulting in an eight-item scale distributed across two domains. This validation process confirmed that the HOPE-P is a valid and reliable tool for assessing expectations in hospitalized patients. Despite these advancements, studies in Spanish on hospitalized patient expectations remain limited. One of the few validated instruments in this language is the Hospitalized Patient Expectations and Received Knowledge Scale (ECPH/CRPH), validated in Spain in a sample of 248 patients undergoing total knee arthroplasty across five public hospitals. This scale consists of 40 items distributed across six dimensions: biophysiological, functional, experiential, ethical, social, and economic. The factorial structure was confirmed in the received knowledge version (CRPH), although in the expectations version (ECPH), a seventh, previously unconsidered dimension emerged [40]. The adaptation of the HOPE-P scale to the Peruvian context is crucial due to cultural, social, and healthcare system differences between China and Peru. Patient expectations are influenced by the local healthcare system, cultural norms regarding physician–patient relationships, and structural barriers to obtaining accurate diagnoses and appropriate treatment. Moreover, in Peru, where healthcare access disparities and cultural diversity are significant, it is essential to ensure that the scale items are culturally relevant and comprehensible for patients. Adapting this scale will allow for a more precise assessment of hospitalized patients’ expectations in Peru, contributing to improving care quality, enhancing patient safety, and reducing disparities in healthcare services. This process will ensure that the HOPE-P scale is a valid and reliable instrument within the Peruvian healthcare system. Therefore, the objective of this study was to adapt and validate the HOPE-P scale in hospitalized Peruvian patients.

## 2. Methods

### 2.1. Design and Participants

The present study is a methodological and cross-sectional investigation [41], which employed convenience sampling. The inclusion criteria considered in this study were being 18 years of age or older, having been hospitalized for a minimum of 24 h, being in adequate cognitive and physical condition to respond to the evaluation, and providing informed consent. It is important to note that, within the gynecology and obstetrics subgroup, women in the active phase of normal labor (uncomplicated dilation) and those admitted solely for physiological childbirth without any additional medical indication were not included. Patients in this department corresponded to gynecological and obstetric cases of a pathological or at-risk nature that required diagnostic and/or therapeutic intervention. In the Peruvian context, any person admitted to a healthcare facility with a medical indication, documented in the clinical record, and a care plan is considered a hospitalized patient—regardless of whether the reason is a disease, an obstetric condition, or a preventive procedure. This definition is supported by the Regulations of the General Health Law of Peru [42] and the current technical standards of the Peruvian Ministry of Health, which stipulate that, upon discharge, the patient must receive a discharge report containing diagnoses, procedures, and recommendations, without distinction by specialty or cause of hospitalization [43]. Therefore, all individuals included in this study met the official definition of a hospitalized patient currently in force in the country.

The minimum sample size was determined using an electronic calculator proposed by Soper [44] taking into account the number of observed and latent variables in the model, an expected effect size (λ = 0.10), a statistical significance level (α = 0.05), and a desired statistical power (1 − β = 0.90). The calculation indicated a minimum of 199 participants, which exceeds the threshold recommended by Kline [45], who suggests at least 200 cases as an acceptable standard for SEM models of moderate complexity. Likewise, the general rule recommending a ratio of ten participants per estimated free parameter was followed, ensuring the stability of the estimates and the reliability of the results [46,47]. In this study, a total of 277 hospitalized patients were recruited, not only meeting the aforementioned criteria but also providing an adequate buffer for potential outliers or data loss. Participants ranged in age from 18 to 85 years (M = 45.87, SD = 17.09), with 59.9% being women. Regarding educational level, the majority had completed secondary education (65.7%). Concerning hospitalization duration, 69.7% of participants had been hospitalized for three to five days. As for the area of care, 56.7% were treated in gynecology and obstetrics, followed by general surgery (28.2%) (Table 1).

### 2.2. Instruments

The Hospitalized Patients’ Expectations for Treatment Scale-Patient Version (HOPE-P) is an instrument developed to assess the treatment expectations of hospitalized patients, covering key aspects of physician–patient communication and expected treatment outcomes. It consists of nine items distributed across two main dimensions: physician–patient communication expectations and treatment outcome expectations. The scale demonstrates high reliability, with a Cronbach’s alpha of 0.761 for the physician–patient communication expectations dimension and 0.919 for the treatment outcome expectations dimension. Each item is rated using a 5-point Likert scale [39].

The Spanish translation of the HOPE-P followed a rigorous cultural adaptation process to ensure linguistic and conceptual fidelity to the original instrument. This process consisted of the following stages:

1. Two native Spanish-speaking bilingual translators independently translated the HOPE-P into Spanish. Both translations were compared to develop an initial consensus version.
2. A committee of experts (including a psychologist, a physician, and the study authors) reviewed the Spanish version, which was then back-translated into English by two native English speakers from the United States, who were fluent in Spanish but unfamiliar with the HOPE-P content. This step ensured that the original meaning of the instrument remained intact.
3. The expert committee reviewed the Spanish version and the back-translated English versions. Based on this evaluation, a preliminary Spanish version of the HOPE-P was developed.
4. The preliminary Spanish version was administered to a focus group of 15 participants to assess its relevance, representativeness, and clarity. No issues were identified during this stage, and no linguistic modifications were necessary.
5. After incorporating all refinements, the final Spanish version of the instrument was developed, officially named *Hospitalized Patients’ Expectations for Treatment Scale-Patient Version*, in Spanish (HOPE-P-S). This final version is presented in Table 2.

### 2.3. Procedure

The study was conducted in strict compliance with ethical principles and was approved by the Ethics Committee of a Peruvian university. The questionnaire was administered in person in common hospitalization areas. Participants were explicitly informed of their inalienable right to withdraw from the study at any time, ensuring their autonomy and guaranteeing that this decision would not result in any negative consequences. Furthermore, the study was rigorously aligned with the guidelines of the Declaration of Helsinki, ensuring that privacy and data confidentiality were protected at all times, adhering to the highest international ethical standards.

### 2.4. Data Analysis

In the preliminary phase, content validity was assessed based on three fundamental aspects for each item: relevance (importance and essentiality for the construct under study), coherence (alignment with the intended construct), and clarity (ease of understanding and unambiguity of the statement). Items were rated on a 0 to 3 scale, where 0 indicated total absence and 3 indicated absolute presence of the evaluated characteristic. To quantify these criteria, Aiken’s V coefficient [48] was calculated with 95% confidence intervals, using custom-developed software in MS Excel©. Aiken’s V ranges from 0 to 1, where values close to 1 indicate a high degree of relevance, coherence, and clarity. Items with an Aiken’s V coefficient ≥ 0.70 were considered acceptable at the sample level, while those whose lower confidence interval exceeded 0.59 were deemed appropriate at the population level [49].

An initial descriptive analysis of the *HOPE-P-S* items was conducted, considering mean, standard deviation, skewness, kurtosis, and corrected item–total correlation. The skewness (g1) and kurtosis (g2) values were deemed acceptable within the range of ±1.5 [50]. Problematic items were identified and excluded using corrected item–total correlation, eliminating those with r(i-tc) ≤ 0.3 or those exhibiting multicollinearity [45].

A confirmatory factor analysis (CFA) was then performed to evaluate the factorial structure of the scale, using the robust maximum likelihood estimation (MLR) method [51]. The model fit indices considered were chi-square (χ^2^), Comparative Fit Index (CFI), and Tucker–Lewis Index (TLI), with expected values ≥ 0.95, as well as Root Mean Square Error of Approximation (RMSEA) and Standardized Root Mean Square Residual (SRMR), which were deemed acceptable at ≤0.08 [45,52]. Additionally, as part of the evidence for internal validity, convergent validity was assessed by calculating the Average Variance Extracted (AVE) for each factor, with an acceptable criterion being a value greater than 0.50 (AVE > 0.50). On the other hand, discriminant validity was evaluated by comparing the AVE of each construct with the square of the inter-factor correlation (ϕ^2^), expecting the AVE to be greater than ϕ^2^ (AVE > ϕ^2^), which indicates adequate empirical differentiation between the factors [53].

The reliability of the scale was assessed using Cronbach’s alpha and McDonald’s omega, with internal consistency considered adequate for values above 0.70 [54].

All statistical analyses were conducted using RStudio [55] with R version 4.1.1 (R Foundation for Statistical Computing, Vienna, Austria; http://www.R-project.org). For confirmatory factor analysis (CFA) and structural equation modeling (SEM), the lavaan package (R version 4.1.1) was used [56].

## 3. Results

### 3.1. Content Validity

Table 3 presents the results of the evaluation of the relevance, representativeness, and clarity of the instrument’s items, using Aiken’s V coefficient and its 95% confidence intervals (CI 95%). Overall, all items achieved Aiken’s V values above 0.80 for each criterion evaluated, indicating a highly positive assessment by experts in terms of relevance, representativeness, and clarity. Items 5, 6, and 8 stood out for obtaining perfect scores (V = 1.00; CI 95%: 0.86–1.00) across all three evaluated criteria, suggesting absolute consensus regarding their clarity, representativeness, and relevance. Additionally, items 3, 4, and 7 also reached the highest score in at least two of the three criteria, demonstrating their robustness within the instrument. On the other hand, item 1 obtained an Aiken’s V of 0.82 for clarity (CI 95%: 0.63–0.93), indicating less unanimity in its clarity rating compared to the other items, although it still falls within an acceptable range. Despite this slight variability, the lower confidence limit of Aiken’s V for all items exceeded the 0.59 threshold, which is the established criterion for an adequate evaluation at the population level.

### 3.2. Descriptive Statistics of Items

The descriptive analysis of the eight items on the HOPE-P-S reveals that the means (M) range from 3.66 (item 7) to 4.01 (item 3), indicating a general tendency toward favorable responses. Item 3 obtained the highest mean, suggesting it was rated most positively by the participants, whereas item 7 had the lowest mean score. The standard deviations (SDs) vary from 0.82 (item 5) to 1.00 (item 2), reflecting a moderate dispersion in responses. Regarding skewness (g1) and kurtosis (g2) values, all fall within the acceptable normality range of ±1.5 [50], suggesting a relatively symmetrical distribution without extreme tails. Specifically, items 1, 3, and 5 show slight negative skewness (g1 values between −0.72 and −0.88), indicating a mild tendency toward higher responses, though without compromising the normality of the data. Corrected item–total correlations (r.cor) range from 0.43 (item 4) to 0.55 (item 5), all above the minimum recommended threshold of 0.30 [45], indicating that each item contributes significantly and coherently to the overall construct assessed by the scale. No items displayed low correlations that would warrant removal. Finally, Cronbach’s alpha remained constant at 0.80 across all internal consistency analyses per item, supporting the instrument’s adequate reliability according to the ≥0.70 results (Table 4).

### 3.3. Confirmatory Factor Analysis

A confirmatory factor analysis (CFA) was conducted based on the two-factor model proposed by Xiao et al. [39] to evaluate the factorial structure of the brief version of the instrument. In the first stage, Model A was tested, consisting of the original eight items distributed across two factors: physician–patient communication (F1) and treatment expectations (F2). The fit indices for this model were acceptable: χ^2^ = 40.059, df = 19, *p* = 0.003; CFI = 0.92; TLI = 0.92; RMSEA = 0.06 (90% CI: 0.04–0.09); SRMR = 0.04. However, item 4, which belonged to the second factor, displayed a low factor loading (λ = 0.41), falling below the acceptable threshold of 0.50 [46], suggesting a limited contribution to the construct. In terms of convergent validity, the physician–patient communication factor showed an Average Variance Extracted (AVE) of 0.51, meeting Fornell and Larcker’s [53] criterion. In contrast, the treatment expectations factor presented a low AVE of 0.40, indicating insufficient convergence among the items in the second factor. Regarding discriminant validity, the inter-factor correlation (φ) between F1 and F2 was 0.52, with a shared variance (φ^2^) of 0.27. While the first factor satisfied the criterion AVE > φ^2^ (0.51 > 0.27), the second factor did not (0.40 < 0.27), limiting the empirical distinction between the two constructs. These results suggest that Model A has limitations in both convergent and discriminant validity, particularly concerning the treatment expectations factor. Subsequently, Model B was tested, excluding item 4 due to its low factor loading. The revised model showed a substantial improvement in fit indices: χ^2^ = 24.980, df = 13, *p* = 0.023; CFI = 0.97; TLI = 0.94; RMSEA = 0.06 (90% CI: 0.03–0.09); SRMR = 0.04. All factor loadings in this model exceeded the minimum threshold of 0.50, supporting the structural validity of the instrument. In terms of convergent validity, there was an increase in AVE values for both factors, physician–patient communication = 0.60 and treatment expectations = 0.55, both meeting the established criterion (AVE > 0.50), indicating that the items appropriately reflect their respective latent constructs. As for discriminant validity, the inter-factor correlation decreased to 0.48, with a shared variance (φ^2^) of 0.23. In this case, both AVE values exceeded the φ^2^ value (0.60 > 0.23 and 0.55 > 0.23), confirming adequate empirical differentiation between the factors. These findings support the internal validity of Model B in terms of both convergent and discriminant validity, reinforcing the decision to remove item 4 to optimize the instrument’s structure (Table 5).

### 3.4. Reliability

Regarding internal consistency, Cronbach’s alpha (α) and McDonald’s omega (ω) coefficients were calculated for both evaluated dimensions. The results were as follows (Table 6).

The obtained coefficients indicate adequate internal consistency for both dimensions, exceeding the 0.70 threshold established by Nunnally and Bernstein [57]. These results support the scale’s reliability, both in its initial version and after the removal of item 4, reinforcing its usefulness in measuring the construct of mental well-being in the proposed context.

## 4. Discussion

The expectations of hospitalized patients significantly impact health outcomes, treatment adherence, and patient safety [1,4]. In surgical contexts, positive expectations enhance recovery and reduce complications [2,5]. In psychiatry and neurology, expectations have been shown to enhance treatment effectiveness [6,9]. The placebo effect plays a crucial role in psychotherapy and dermatology, influencing treatment efficacy [10,12], though its impact is less significant in dental prosthetics and fracture recovery [14]. Patients with low expectations tend to exhibit lower adherence to treatment, whereas unrealistic expectations can lead to treatment dissatisfaction [17,18]. Expectations also affect the perception of side effects, such as anticipated nausea during chemotherapy, which can increase the severity of symptoms [20]. Moreover, physicians’ expectations influence quality of care and clinical outcomes [22]. Positive expectations from physicians have been found to enhance neurochemical responses and reduce stress [3,29]. To effectively measure expectations, the HOPE-P scale evaluates both the physician–patient relationship and treatment expectations, demonstrating high validity [39]. Its adaptation to the Peruvian context will allow for more accurate assessments of patient expectations, ultimately improving healthcare quality and patient outcomes.

The present study aimed to validate the HOPE-P-S through a Confirmatory Factor Analysis (CFA), based on the two-factor model proposed by Xiao et al. [39]. Overall, the findings align with those reported by the original authors regarding the bifactorial structure of the scale, supporting the model’s stability across different linguistic and cultural contexts. In the first stage, Model A was evaluated, consisting of the original eight items distributed across two factors: (1) expectations of physician–patient communication (items 1–3) and (2) expectations regarding treatment outcomes (items 4–8). The model fit indices were acceptable, preliminarily confirming the adequacy of the bifactorial model. However, item 4 showed a low factor loading (λ = 0.41), below the acceptable threshold of 0.50, which justified its removal from the model. This item, which assessed the expectation of receiving a definitive diagnosis, exhibited weak psychometric performance, possibly due to cultural differences in the perception of diagnostic certainty. Its inclusion reduced the internal consistency of the second factor, suggesting that its content may represent a distinct conceptual domain that requires separate assessment. Regarding convergent validity, the analysis of Average Variance Extracted (AVE) showed that only the first factor (physician–patient communication) reached an acceptable value (AVE = 0.51), whereas the second factor (treatment expectations) did not meet the recommended minimum criterion (AVE = 0.40), indicating that the items of the latter factor did not sufficiently explain the construct’s variance. Concerning discriminant validity, the inter-factor correlation was φ = 0.52, implying a shared variance of φ^2^ = 0.27. While the AVE for the first factor was greater than the shared variance (0.51 > 0.27), the second factor did not meet this condition (0.40 < 0.27), suggesting insufficient empirical differentiation between the two constructs. These findings limit the internal validity of Model A, particularly regarding the second factor. In light of these results, an adjusted model (Model B) was evaluated, excluding item 4. This modification significantly improved the fit indices, and all factor loadings exceeded the minimum value of 0.50, supporting the internal consistency and structural validity of the scale. From the perspective of convergent validity, both factors reached satisfactory AVE values (physician–patient communication = 0.60; treatment expectations = 0.55), exceeding the 0.50 threshold proposed by Fornell and Larcker [53], indicating that the items adequately explained the variance of their respective constructs. Likewise, discriminant validity was also confirmed in this model. The inter-factor correlation decreased to φ = 0.48 (φ^2^ = 0.23), and both the AVE for the first factor (0.60) and the second (0.55) were greater than the shared variance, thereby meeting the criterion for empirical differentiation between factors (AVE > φ^2^). These results support the bifactorial structure of the instrument and strengthen its internal validity. In summary, the removal of item 4 improved both the convergent and discriminant validity of the instrument and optimized the model fit. Therefore, the use of Model B is recommended as the most parsimonious and psychometrically robust version of the HOPE-P-S for hospitalized Spanish-speaking populations.

Internal reliability of the HOPE-P-S was also assessed using Cronbach’s alpha (α) and McDonald’s omega (ω) coefficients in both models. The values obtained for Model B ranged from 0.76 to 0.77 across both dimensions of the bifactorial model. These values exceed the minimum recommended threshold of 0.70 for instruments under development [57] indicating acceptable internal consistency. When comparing these results with those reported by Xiao et al. [39]. for the original English version (HOPE-P), a similarity is observed in the physician–patient communication expectations dimension (α = 0.761 in the original vs. α = 0.76 in this adaptation). However, the treatment expectations subscale in our sample yielded a noticeably lower coefficient (α = 0.77) compared to the value reported in the original version (α = 0.919). This discrepancy may be attributed to several contextual and cultural factors. First, the perception of treatment outcomes may vary significantly depending on the healthcare system in which patients are situated. In environments characterized by greater diagnostic uncertainty, barriers to access, or delays in care, treatment expectations may be more heterogeneous or less stable, potentially affecting response consistency. Second, cultural differences in the expression of expectations and trust in the healthcare system may also influence how patients interpret and respond to items related to treatment outcomes. Additionally, the process of translation and cultural adaptation of the instrument may introduce subtle semantic shifts in certain items, which could impact their factor loadings or their overall contribution to internal consistency. This is particularly relevant considering that no items were removed in the original model, whereas item 4 had to be excluded in our version due to a low factor loading. On the other hand, although the original version reported a test–retest reliability of 0.670 (*p* = 0.001) after seven days, temporal stability has not yet been evaluated in this version. It is recommended that future longitudinal studies assess this property and examine the replicability of scores over time, in order to strengthen the evidence for reliability in Spanish-speaking contexts.

### 4.1. Implications

The adaptation and validation of the HOPE-P-S has a significant impact on clinical practice. Having a valid and reliable instrument enables healthcare professionals to more accurately assess patients’ expectations, a crucial factor that influences treatment satisfaction, therapeutic adherence, and ultimately, clinical outcomes. These findings highlight the importance of promoting effective communication between physicians and patients, encouraging a patient-centered approach that facilitates shared decision-making. In hospital settings, the systematic incorporation of the HOPE-P-S during initial assessments could help medical teams identify discrepancies between patient expectations and potential treatment outcomes, allowing for interventions aimed at aligning these expectations, enhancing the hospital experience, and reducing the risk of dissatisfaction and treatment abandonment.

At the health policy level, this study supports the inclusion of standardized tools like the HOPE-P-S in patient-centered care protocols. Health authorities could advocate for its implementation in national quality improvement programs, particularly in high-demand settings such as public hospitals and rural clinics. The results suggest that expectation alignment not only enhances clinical outcomes but also optimizes resource utilization by reducing complications and prolonged hospital stays. Moreover, adapting the HOPE-P-S to the Peruvian context underscores the importance of considering cultural and social specificities in health policy design. Therefore, it is recommended that institutions provide training for medical staff in effective communication skills and cultural awareness, integrating these as quality assessment criteria in hospital audits.

This study enriches the theoretical framework on expectations in healthcare, confirming their relevance as a multidimensional construct encompassing both communicative factors and expected treatment outcomes. The findings reinforce the applicability of the biopsychosocial model in analyzing physician–patient interactions, emphasizing how expectations affect not only psychological aspects of the patient but also their physiological response to treatment. From a methodological perspective, the results support the use of techniques such as Confirmatory Factor Analysis (CFA) to ensure the structural robustness of culturally adapted instruments. The elimination of problematic items also highlights the need to adapt existing tools to ensure their relevance and accuracy in different cultural contexts. These findings could guide future studies exploring the impact of expectations in less-explored fields, such as primary care or palliative treatments, contributing to a more comprehensive understanding of how expectations shape healthcare experiences.

### 4.2. Limitations

Despite the significant contributions of this study to the adaptation and validation of the HOPE-P-S in the Peruvian context, it is important to acknowledge certain limitations that may have influenced the results and should be considered in future research. First, the cross-sectional design employed prevents the assessment of the instrument’s temporal stability. The absence of a test–retest analysis limits the ability to determine the consistency of measurements over time, which is essential for confirming its reliability in dynamic clinical settings. Future studies should incorporate longitudinal follow-ups to evaluate the evolution of patient expectations and the stability of the instrument. Another important limitation lies in the sampling method employed. Convenience recruitment in two hospitals in Lima may have introduced selection bias, thereby limiting the generalizability of the findings to other hospital settings and regions of the country. Future studies are encouraged to expand the sample to include facilities from different regions and levels of care to enhance representativeness. Furthermore, the overrepresentation of patients from the gynecology and obstetrics department, including those admitted for childbirth, may have influenced the results, as their expectations and experiences could differ from those of patients hospitalized for illnesses or therapeutic interventions. Therefore, subsequent research should aim for a more balanced sampling across specialties, distinguishing these subgroups and conducting comparative analyses to identify variations in responses and item functioning, thus strengthening the construct’s validity and applicability. Additionally, the exclusive use of self-report measures may have been subject to social desirability bias or temporary emotional conditions, potentially affecting response accuracy. Incorporating semi-structured interviews or additional data sources would allow for a more comprehensive assessment of the construct. Finally, a relevant conceptual limitation is that, like the original version, the HOPE-P-S focuses exclusively on expectations toward the attending physician, without accounting for the patient’s interaction with other healthcare professionals. This restriction may limit full capturing the “treatment expectations” construct in multidisciplinary hospital contexts. Therefore, future studies are encouraged to develop complementary subscales that incorporate expectations toward other clinical actors and to conduct exploratory qualitative research to expand understanding of the phenomenon.

## 5. Conclusions

The present research makes a significant contribution to the healthcare field by adapting and validating the HOPE-P-S, offering a psychometrically robust and culturally relevant tool for assessing treatment expectations in hospitalized Peruvian patients. The empirical evidence obtained—including a strong factorial structure, adequate reliability and validity indices, and sensitivity to the local clinical and sociocultural context—reinforces the utility of this instrument in improving physician–patient communication, promoting shared decision-making and optimizing clinical outcomes through effective expectation alignment. In doing so, it enhances not only the quality of hospital care but also patient safety and the efficiency of healthcare resource utilization. However, the use of a cross-sectional design, convenience sampling, and the exclusive focus on the attending physician constitute limitations that restrict the generalizability and comprehensive understanding of the phenomenon within multidisciplinary teams. Future studies should address these limitations through longitudinal designs, nationally representative samples, and the inclusion of expectations toward other healthcare professionals. Additionally, it is recommended to explore the longitudinal impact of expectations on treatment adherence, clinical progress, and subjective well-being.

## Figures and Tables

**Table 1:** Sociodemographic characteristics.

Characteristics		*n*	%
Gender	Female	166	59.9
	Male	111	40.1
Educational Level	Primary	51	18.4
	Secondary	182	65.7
	Higher Education	44	15.9
Hospitalization Duration	3 to 5 days	1	0.4
	1 to 2 days	47	17
	3 to 5 days	193	69.7
	More than 7 days	14	5.1
	Less than 1 day	22	7.9
Hospitalization Area	Gynecology and Obstetrics	158	56.7
	General Surgery	78	28.2
	Thoracic and Cardiovascular Surgery	14	5.1
	Traumatology	9	3.2
	Internal Medicine	18	6.5
Socioeconomic Level	Middle Class	56	20.2
	Extreme Poverty	36	13
	High Income Level	5	1.8
	Moderate Poverty	180	65

**Table 2:** Spanish adaptation of the Hospitalized Patients’ Expectations for Treatment Scale.

HOPE-P	*HOPE-P-S*
Subscale A: doctor–patient communication expectation	A: Expectativa de comunicación médico-paciente
Q1. The doctor listens to my opinions on treatment	El médico escucha mis opiniones sobre el tratamiento.
Q2. During this hospitalization, the doctor fully explains the state of illness to me and negotiates medical decisions with me	Durante esta hospitalización, el médico explica completamente el estado de mi enfermedad y negocia decisiones médicas conmigo.
Q3. During this hospitalization, the doctor is caring	Durante esta hospitalización, el médico muestra cuidado y preocupación.
Subscale B: treatment outcome expectation	B: Expectativa sobre el tratamiento
Q4. Through this hospitalization, the disease can be definitely diagnosed	Durante esta hospitalización, la enfermedad puede ser diagnosticada definitivamente
Q5. Through this hospitalization, symptoms can be improved	Durante esta hospitalización, los síntomas pueden mejorar
Q6. Through this hospitalization, the disease can be cured	Durante esta hospitalización, la enfermedad puede ser curada
Q7. Through this hospitalization, I can restore work/family functions	Durante esta hospitalización, puedo restaurar las funciones laborales/familiares
Q8. Through this hospitalization, I can take care of myself	Durante esta hospitalización, puedo cuidar de mí mismo

**Table 3:** Aiken’s V coefficient.

	Relevance				Representativeness				Clarity			
Items	M	DE	V	CI 95%	M	DE	V	CI 95%	M	DE	V	CI 95%
1	2.92	0.28	0.97	(0.82–1.00)	2.92	0.28	0.97	(0.82–1.00)	2.46	0.52	0.82	(0.63–0.93)
2	2.92	0.28	0.97	(0.82–1.00)	2.85	0.38	0.95	(0.78–0.99)	2.77	0.44	0.92	(0.75–0.98)
3	3.00	0.00	1.00	(0.86–1.00)	2.92	0.28	0.97	(0.82–1.00)	2.85	0.38	0.95	(0.78–0.99)
4	3.00	0.00	1.00	(0.86–1.00)	3.00	0.00	1.00	(0.86–1.00)	2.92	0.28	0.97	(0.82–1.00)
5	3.00	0.00	1.00	(0.86–1.00)	3.00	0.00	1.00	(0.86–1.00)	3.00	0.00	1.00	(0.86–1.00)
6	3.00	0.00	1.00	(0.86–1.00)	3.00	0.00	1.00	(0.86–1.00)	3.00	0.00	1.00	(0.86–1.00)
7	3.00	0.00	1.00	(0.86–1.00)	3.00	0.00	1.00	(0.86–1.00)	2.92	0.28	0.97	(0.82–1.00)
8	3.00	0.00	1.00	(0.86–1.00)	3.00	0.00	1.00	(0.86–1.00)	3.00	0.00	1.00	(0.86–1.00)

Note: M = media; DE = Desviación Estándar; V = Aiken’s V.

**Table 4:** Descriptive statistics.

Items	M	SD	g1	g2	r.cor	α
1	3.94	0.89	−0.88	0.9	0.48	0.8
2	3.79	1	−0.68	−0.15	0.52	0.8
3	4.01	0.89	−0.72	0.1	0.53	0.8
4	3.67	0.94	−0.6	−0.03	0.43	0.8
5	3.91	0.82	−0.77	0.73	0.55	0.8
6	3.75	0.93	−0.45	−0.49	0.54	0.8
7	3.66	0.92	−0.48	−0.25	0.5	0.8
8	3.7	0.97	−0.67	0.03	0.47	0.8

**Table 5:** Confirmatory factor analysis.

Items	Model A		Model B	
	λ (F1)	λ (F2)	λ (F1)	λ (F2)
1	0.76		0.76	
2	0.65		0.64	
3	0.75		0.75	
4		0.41		
5		0.66		0.65
6		0.74		0.73
7		0.69		0.71
8		0.61		0.62
AVE	0.51	0.4	0.6	0.55
φ^2^		0.27		0.23
φ	0.52		0.48	

Note: F1 = physician–patient communication; F2 = treatment expectations; λ = standardized factor loading; AVE = Average Variance Extracted; φ = below the diagonal: inter-factor correlations; φ^2^ = above the diagonal: shared variance between factors.

**Table 6:** Model reliability.

Reliability	Model A	Model B
Physician–Patient Communication Expectation	α = 0.76; ω = 0.76	α = 0.76; ω = 0.76
Treatment Outcome Expectation	α = 0.75; ω = 0.76	α = 0.77; ω = 0.77

## Data Availability

The data that support the findings of this study are available from the corresponding author upon reasonable request.
